# Analyzing Response Rates and Respondent Demographics in Online Surveys Assessing Public Perception of Alzheimer’s Disease‐Modifying Therapies

**DOI:** 10.1002/alz.084862

**Published:** 2025-01-09

**Authors:** Saki Nakashima, Kenichiro Sato, Yoshiki Niimi, Tatsushi Toda, Takeshi Iwatsubo

**Affiliations:** ^1^ The University of Tokyo, Tokyo Japan

## Abstract

**Background:**

With the recent approval of lecanemab, a disease‐modifying therapy (DMT) for Alzheimer’s Disease (AD), there is an urgent need to address healthcare system challenges to ensure the safe, effective, and sustainable delivery of approved DMT drugs.

Understanding public perceptions of these challenges through online surveys might be helpful. Achieving sufficient responses that reflect the characteristics of the respondents is crucial for a successful online survey, however, details about the respondent characteristics for this kind of survey are largely uncertain.

**Method:**

We analyzed data from an anonymous online survey conducted using Google Forms from November 28 to December 16, 2023 to participants of a Japanese trial‐ready cohort (J‐TRC) web study, a web‐based online registry aiming to enroll individuals with preclinical AD to facilitate AD prevention trials.

**Result:**

Among approximately 10,400 J‐TRC web study participants to whom we sent invitation e‐mails, we received 1,761 responses (16.9% response rate) within two weeks of the response acceptance period. The demographic split of survey respondents was 47% male and 52% female, primarily between the ages of 50‐70. Male respondents were more predominant in older age groups (Chi‐squared p<0.001). Regarding the days of the week when invitation e‐mails were sent (Tuesday to Saturday), Tuesday had the highest response rate, followed by Saturday, Wednesday, Thursday, and Friday. Female respondents were more predominant on Wednesday, Friday, and Saturday. Respondents from Tuesday to Friday were more likely to be retirees, whereas those on Saturday were not. The effect of retirement on increasing response rates was more pronounced in males than in females.

**Conclusion:**

Our analysis evaluated the response rate and respondent characteristics for an online survey concerning public perceptions of DMTs for AD. The current results could be useful for future efforts to improve response rates of online surveys targeting health‐conscious individuals aged 50‐80 with an interest in dementia treatment, by optimizing the timing and methods of sending survey invitations.

